# Supplementary material for: Genetic diversity among maize (Zea mays L.) inbred lines adapted to Japanese climates
Source: PLoS One. 2024 Jan 25;19(1):e0297549. doi: 10.1371/journal.pone.0297549 (PMC10810424; doi:10.1371/journal.pone.0297549)
Supplement: Attachment — Submitted filename: 231107 Letter for editor.docx [file pone.0297549.s003.docx]

Thank you very much for providing many useful insights.

We have reflected these comments described below.

Reviewer #1: This manuscript is well written.
However, I am suggesting a few corrections:
Line 21 - It is not clear what you mean by 'Self-pollinated and open-pollinated' inbred lines. Conventionally, inbred lines are developed by self pollination and maintained by self pollination. Open pollination means there is no control of the pollen parent. I have an idea what you mean by 'open pollinated inbred lines ' because I have read the whole manuscript, but in the abstract the meaning has to be very clear.

　→We guess this point is very important and have corrected Abstract, according to the detailed description in the main text.

“A self-pollinated line derived from a classic F_1_ variety and another line from an open-pollinated population from the same derivation were classified as separate components by PCA and Bayesian clustering.”

Line 74 - Change the sentence to 'DNA was extracted as previously described by Tamaki et al. [ 10 ].'

Line 199 - The phrase 'were classified as other components' may be changed to 'were classified as separate components'

→We modified the expression you pointed out.

We have also reflected on other points raised in another paper of ours regarding inbred names. We have also noted them in the text,

↓(L63-)

“Ki” is used as the name of a breeding line in Nagano Pref. and the first letter for the Kikyogahara area in Shiojiri, Nagano Pref., but it is also the name of an inbred line at Kasetsart University in Thailand [11]. To avoid confusion, we have used conservation names starting with “J” and “JC” instead of “Ki”

In accordance with this description, all applicable “Ki” inbred names were corrected to the conservation number (J- or JC-) in the main text, Tables and Figures.

Table 1 etc.

We changed “KOARC, NARO” to “KARC, NARO”. It was our careless mistake.

L132-

The text was changed because the interpretation of the PCA results for 'Na94' was wrong. Also, MIS in Figure 2 is shown in red shape to make the classification visually clearer. You could have a clear picture of the three and two separated MIS.

L213-

To gain more insight into the research, we added the following description.

“Since the origin of these inbred lines especially before the 1990s, is based on old handwritten records, further details, including the possibility of human error, should be examined in the future.”

The English text has been reviewed again by a specialist and the resulting corrections have been reflected.
