# Supplementary material for: Genetic diversity among maize (Zea mays L.) inbred lines adapted to Japanese climates
Source: PLoS One. 2024 Jan 25;19(1):e0297549. doi: 10.1371/journal.pone.0297549 (PMC10810424; doi:10.1371/journal.pone.0297549)
Supplement: Attachment — Submitted filename: 231201 Response to Reviewers.docx [file pone.0297549.s004.docx]

Thank you very much again for providing many useful insights.

We have reflected these comments described below.

Reviewer #1: The manuscript has been improved, however, I am suggesting a few more corrections.

Line 16

The phrase ‘vital to developing’ should be changed to ‘vital for development of’

Line 32

Change ‘raising food for self-sufficiency’ to ‘raising food production for self-sufficiency’

Lines 44-46

Change sentence to ‘…. for the development of superior F1 varieties ….,. and will enable the classification of inbred lines into heterotic groups ….’

Line 63

Put a comma before the word ‘respectively’.

Line 187

Change the phrase ‘than that in’ to ‘than those between’

Line 205

Delete the words ‘that of’

→We modified all the expressions you pointed out.

Also, in the tables, all relevant 'Ki' inbred names that were lost to change were corrected to their conservation numbers (J- or JC-).

And other minor corrections were made.

Journal Requirements:

Please review your reference list to ensure that it is complete and correct. If you have cited papers that have been retracted, please include the rationale for doing so in the manuscript text, or remove these references and replace them with relevant current references. Any changes to the reference list should be mentioned in the rebuttal letter that accompanies your revised manuscript. If you need to cite a retracted article, indicate the article’s retracted status in the References list and also include a citation and full reference for the retraction notice.

→We have confirmed the reference list carefully.
